# Telepathology Development and Stakeholder Perspectives in China: Cross-Sectional Survey

**DOI:** 10.2196/83514

**Published:** 2026-03-16

**Authors:** Linlin Wang, Fangfang Cui, Xianying He

**Affiliations:** 1 National Engineering Laboratory for Internet Medical Systems and Applications, The First Affiliated Hospital of Zhengzhou University, 1 Jianshe Road, Zhengzhou, Henan, 450052, China, 86 037167966286, 86 037167966286; 2 Shanghai Artificial Intelligence Laboratory, Shanghai, China

**Keywords:** telepathology, digital slide scanners, software, service, usage, satisfaction, perceptions

## Abstract

**Background:**

Telepathology is widely recognized for improving diagnostic access in remote areas and is strongly promoted in China to address the shortage of pathologists. However, a comprehensive national assessment of its implementation and multistakeholder perspectives remains lacking.

**Objective:**

This study aims to conduct a comprehensive analysis of the infrastructure, practical application, and user experience of telepathology in China from 2018 to 2023 and to identify the key factors affecting its adoption and satisfaction levels.

**Methods:**

A repeated cross-sectional survey was administered to hospitals across China (2018-2023), alongside a parallel survey of telepathology users (2023). Data were analyzed from hospitals (sample sizes ranging from 464 to 188 across years), pathologists (n=262), and patients (n=141). Regression analysis was applied to investigate factors associated with professional satisfaction and willingness to adopt the technology.

**Results:**

The proportion of hospitals with telepathology software grew at an annual average rate of 7.60%, increasing from 29.5% (137/464) in 2018 to 42.6% (80/188) in 2023. The median number of telepathology cases per hospital increased from 51 to 200, with the fastest growth observed in the eastern region. Among pathologists, 61.1% (160/262) used telepathology weekly, 61.5% (161/262) spent 11 to 20 or 21 to 30 minutes. Pathologists reported a 95.8% (251/262) satisfaction rate, and 95% (249/262) were willing to continue participation. Convenience of system operation and service processes were associated with enhanced satisfaction and sustained engagement willingness (*P*<.05). Among patients, 92.2% (130/141) received diagnostic results within 24 hours, with 100% (141/141) satisfaction, and 99.3% (140/141) were willing to recommend telepathology.

**Conclusions:**

Telepathology in China grew significantly from 2018 to 2023, with high satisfaction among both pathologists and patients. For sustained development, a multilevel approach is essential: improving system usability, implementing supportive policies (eg, funding and insurance coverage), and conducting targeted promotion of its proven benefits. This integrated strategy is key to making telepathology a sustainable solution for equitable diagnostic access nationwide.

## Introduction

Pathological diagnosis is the gold standard for disease diagnosis, particularly for cancer. However, China’s primary medical institutions face a significant shortage of pathologists, especially experienced ones. According to the 2019 China Pathology Quality Report [1], there are only 0.55 pathologists per 100 hospital beds, and 46.3% of secondary hospitals have just 1 pathologist, which is far below the demand for timely and high-quality pathology services. Data from the Pathology Quality Control Center of China [2] reveal that 82% of senior pathologists are concentrated in tertiary hospitals, leaving many primary hospitals without senior-title pathologists. In central and western regions, such as Tibet, Xinjiang, Qinghai, and Guizhou, more than 20% of institutions have no more than 1 pathologist, highlighting a significant imbalance in pathology resource distribution across China.

Telepathology, first proposed in 1987 [3], addresses resource shortages by enabling the transmission of static images, dynamic videos, and whole-slide images (WSIs). As defined in the Basic Functions Specification of Telemedicine Information System (WS/T529-2016), telepathology involves the submission of diagnostic requests and patient data by an inviting party to an invited expert, who then provides diagnostic opinions and reports. Central to this practice is WSI technology, which converts pathological slides into high-resolution, zoomable digital images and supports the diagnosis of all pathology-dependent diseases, including tumor classification and grading, as well as the identification of specific pathogens or special inflammatory lesions. Its diagnostic reliability is supported by multiple guidelines and studies. The College of American Pathologists (CAP) released relevant validation guidelines in 2013 and 2021 [4,5], and the Royal College of Pathologists issued “Best Practice Recommendations for Implementing Digital Pathology” [6]. Studies by Montgomery et al [7] and Shinohara et al [8] confirmed the high diagnostic accuracy and effectiveness of WSIs in remote diagnosis. Furthermore, Girolami et al’s [9] meta-analysis validated the diagnostic concordance between WSIs and light microscopy, reinforcing its role in delivering high-quality diagnostic services to resource-limited regions. At the regulatory and application levels, pathological diagnosis based on WSIs has been approved by the Food and Drug Administrations of the United States, the European Union, and Japan [10-12]. Since the US Food and Drug Administration first authorized a WSI system for primary diagnosis in 2017, multiple integrated digital pathology platforms, combining scanning, storage, viewing, and analysis functions, have received 510(k) clearance [13]. As of December 31, 2025, China’s National Medical Products Administration (NMPA) had also approved 71 digital pathology–related medical devices [14]. In practice, WSI-based telepathology has been widely adopted in numerous countries and regions, including Europe [15], Nigeria [16], India [17], Tanzania [18], and Canada [19].

In China, developing a WSI-based telepathology system is a core strategy to address the deficiencies in grassroots pathology services [20]. Preliminary studies have demonstrated the effectiveness of the national cloud-based telepathology platform [21] and its potential to optimize resource allocation [22]. However, the existing literature lacks a comprehensive, long-term, and systematic evaluation of the overall development of telepathology in China. Key questions remain unanswered: What are the national trends and regional disparities in its adoption? As primary users, what are the adoption behaviors, perceptions, and influencing factors among pathologists? Moreover, what are patients’ experiences and satisfaction levels with telepathology services? The absence of such critical information limits a thorough understanding of current implementation barriers and hinders targeted interventions.

To address these gaps, this study utilizes nationwide survey data from 2018 to 2023 with the following objectives: (1) to delineate the developmental trends and spatial distribution characteristics of telepathology in China over the past 6 years; (2) to analyze, from the perspective of pathologists, their usage behaviors, attitudes, and key influencing factors; and (3) to conduct the first systematic assessment of patient satisfaction with telepathology services. By integrating multidimensional evidence, including macro-level trends, insights from pathologists, and patient experiences, this study aims to provide an empirical foundation and decision-making support for optimizing China’s telepathology infrastructure and promoting the equitable distribution of high-quality pathology resources.

## Methods

### Study Setting

To understand the current status of and identify obstacles to telemedicine services (telemedicine service items include teleconsultation, telepathology, teleradiology, telemonitoring, tele-outpatient services, etc), the Telemedicine Informationization Professional Committee of China (TIPC) and the National Telemedicine Center of China (NTCC) have conducted web-based surveys on telemedicine since 2018.

### Survey on the Development of Telemedicine in Hospitals

This survey targeted hospitals approved by Chinese health administrative departments. The questionnaire was developed by experts with more than 5 years of telemedicine experience and refined annually based on prior findings. Following the initial design, 10 to 18 hospitals were selected for pilot testing, and feedback was incorporated to finalize the questionnaire.

### Survey on the Usage and Perceptions of Telemedicine

A survey was conducted among doctors and patients participating in telemedicine services. The questionnaires were designed through needs analysis, a review of telemedicine practices, and relevant literature. After drafting, a pilot test was administered at the Henan Provincial Telemedicine Center and selected subcenters. The instruments were further optimized based on pilot feedback to form the final versions.

### Implementation of the Survey

The electronic open survey was an online questionnaire on NTCC’s website [23]. The survey announcement was formally issued by TIPC via an official red-headed document. Each annual theme had its own webpage, with up to 42 mandatory questions (the exact number depended on the respondent’s answers) across 18 to 25 pages in Chinese. Completion required approximately 8 to 12 minutes based on pilot testing. The surveys were open from July to September 2018, October to November 2019, September to October 2020, and September to October 2023. To ensure data quality, duplicate submission prevention was implemented through IP address verification, response-time logic control, and cross-validation with official hospital records. Response metrics for each questionnaire type were as follows:

- The hospital questionnaire recorded 896 views with a 63.3% (567/896) participation rate and an 81.8% (464/567) completion rate in 2018
- The corresponding figures for 2019, 2020, and 2023 were 482 views (60.8% participation, 82.6% completion), 401 views (61.1% participation, 83.3% completion), and 364 views (63.2% participation, 81.7% completion).
- The doctor questionnaire received 3269 views, achieving a 53.1% (1736/3269) participation rate and a 77.9% (1353/1736) completion rate, including 262 pathologists among the completers.
- The patient questionnaire garnered 3528 views, with a 42.6% (1502/3528) participation rate and a 59.3% (891/1502) completion rate, of whom 141 patients had used telepathology services.

### Data Cleaning and Valid Questionnaire

After the collection deadline, data were exported to Excel for cleaning. Hospital questionnaires from identical or conflicting IP addresses that could not be verified against official hospital records (eg, outpatient visits, bed counts) were excluded. Additionally, hospital questionnaires completed in less than 2 minutes and multiple doctor or patient questionnaires submitted from the same IP address within a 3-minute interval were omitted. Valid hospital questionnaires totaled 464 in 2018, 242 in 2019, 204 in 2020, and 188 in 2023.

### Study Design

To explore the current status of telepathology development and application in Chinese hospitals, this study used data from TIPC and NTCC. Surveys were conducted in 2018, 2019, 2020, and 2023 across eastern, central, and western China. Due to the COVID-19 pandemic, restrictions on personnel movement during the outbreak disrupted normal work routines and medical services, resulting in the cancellation of surveys that were planned for 2021 and 2022. The eastern, central, and western regions are classified according to standards set by the National Bureau of Statistics of China (Multimedia Appendix 1). The survey coverage area is presented in Multimedia Appendix 2. Pearson chi-square test indicated no significant difference in provincial distribution among these regions across the 4 years (*P*=.90). Telepathology-related data were selected to assess construction progress and development trends. Based on 2023 survey responses from pathologists and patients who participated in telepathology services, application effectiveness was further analyzed.

This study was reported in accordance with the CHERRIES (Checklist for Reporting Results of Internet E-Surveys) guidelines (Checklist 1) [24]. The hospital questionnaire included 11 repeated survey questions on telepathology, comprising 9 single-choice questions and 2 fill-in questions. Among them, 6 questions related to telepathology infrastructure and service volume, and 5 questions related to telepathology operations and billing. The questionnaire was completed by telemedicine organizers at each hospital. The doctor questionnaire consisted of 24 questions, including basic information (7 questions), telepathology service usage and experience (9 questions), evaluation and attitudes (6 questions), and open-ended questions regarding problems and expectations (2 questions). The patient questionnaire consisted of 19 questions, including basic information (7 questions), usage (7 questions), and evaluation and attitudes (5 questions) (Multimedia Appendix 3).

### Data Analysis

Data cleaning was performed to avoid logical problems and ensure data quality. Descriptive statistics were used to analyze the data. Median values and IQR are reported for quantitative data, and percentages are reported for qualitative data. Chi-square tests and Fisher exact probability tests were used to assess differences. Variables selected through univariate analysis were further analyzed using multivariate ordinal logistic regression to identify factors influencing satisfaction and usage intention [25]. All statistical analyses were conducted using SPSS (version 20.0; IBM Corp). All tests were 2-tailed, and *P*<.05 was considered statistically significant. Chinese maps were generated using ArcMap (version 10.8.1; Esri Corp) and R language (version 4.4.1; R Foundation for Statistical Computing).

### Ethical Considerations

This study was approved by the institutional review board at the First Affiliated Hospital of Zhengzhou University (protocol 2021-KS-HNSR181). Ethics approval was obtained with the stipulation that collected data must not expose respondents’ identities and only aggregated information would be addressed in the research paper. Informed consent was obtained from all participants. The survey was conducted without compensation. The questionnaire was anonymous, and the data file was stored on the NTCC intranet server. Only research team members who signed confidentiality agreements were permitted to access the data.

## Results

### The Construction of Telepathology

**Figure 1.**
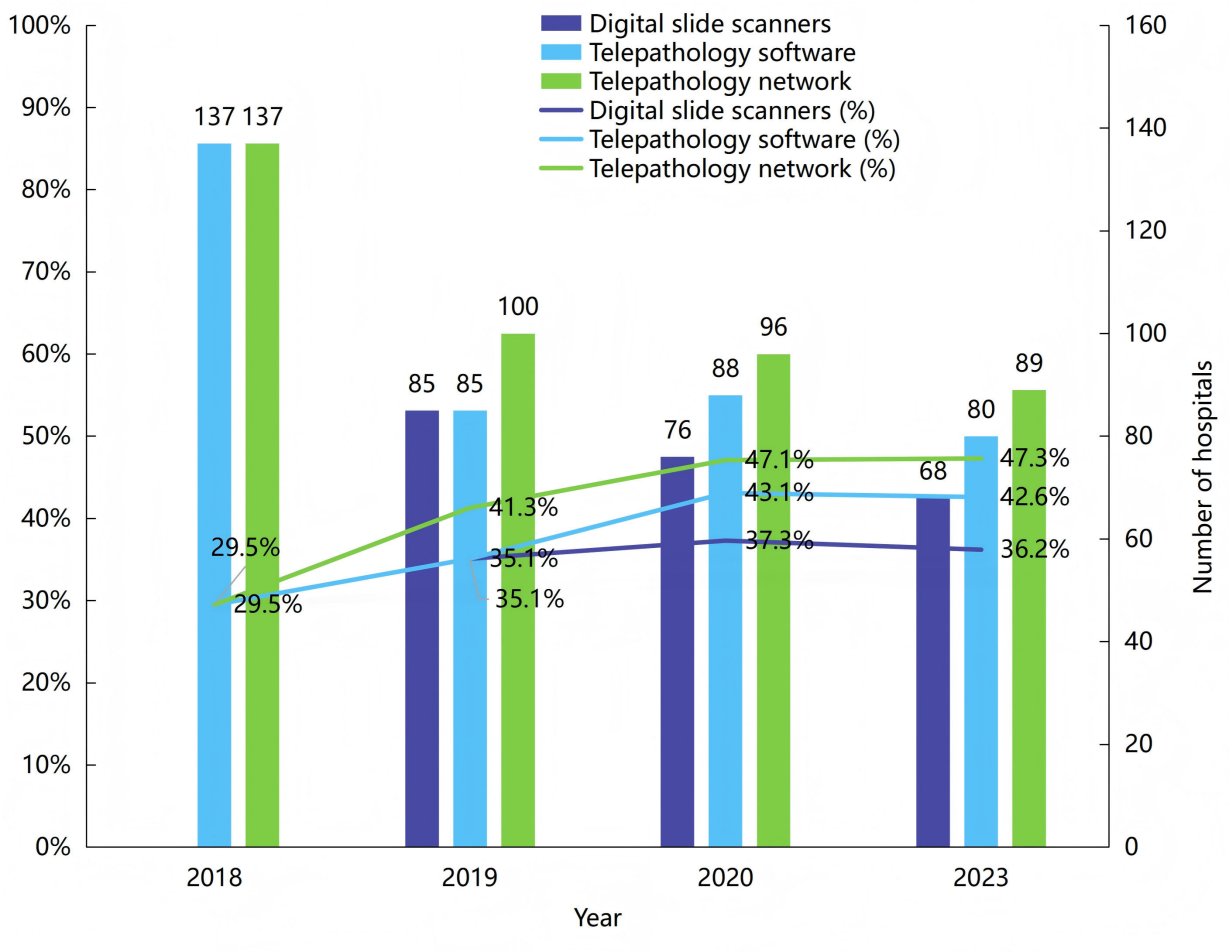
In 2018‐2020 and 2023, the construction of telepathology infrastructure.

Digital slide scanners, software, and networks were investigated, as shown in Figure 1. The configuration status of digital slide scanners was not investigated in 2018. In 2019, 2020, and 2023, the number of hospitals equipped with scanners were 85 of 242 (35.1%), 76 of 204 (37.3%), and 68 of 188 (36.2%), respectively, with an annual average growth rate of 0.75%. From 2018 to 2023, the percentages of hospitals deploying telepathology software were 29.5% (137/464), 35.1% (85/242), 43.1% (88/204), and 42.6% (80/188), respectively, with an annual average growth rate of 7.6%. The telepathology service network comprises private networks and the internet. In 2018, 2019, 2020, and 2023, there were 137 (137/464, 29.5%), 100 (100/242, 41.3%), 96 (96/204, 47.1%), and 89 (89/188, 47.3%) hospitals connected to the telepathology service network, respectively. Private networks were consistently more utilized than the internet, with annual ratios of 68.6% (94/137) vs 31.4% (43/137), 74% (74/100) vs 26% (26/100), 67% (64/96) vs 33% (32/96), and 93% (83/89) vs 7% (6/89).

### The Current Status of Telepathology Services

In 2018, 2019, 2020, and 2023, the proportions of hospitals providing telepathology services were 23.3% (108/464), 28.9% (70/242), 33.3% (68/204), and 31.4% (59/188), respectively, with an annual average growth rate of 6.1%. Among hospitals with established telepathology systems, the proportions providing telepathology services were 78.8% (108/137) in 2018, 82.3% (70/85) in 2019, 77.3% (68/88) in 2020, and 73.8% (59/80) in 2023. The median numbers of telepathology cases for these years were 51 (IQR 10‐200), 95 (IQR 18.25‐228.5), 100 (IQR 27.5‐223.75), and 200 (IQR 93‐331.5), respectively. A statistically significant increase was observed from 2018 to 2023, with an annual average growth rate of 31.4%. Table 1 details the regional and hospital-level distribution. From 2018 to 2020, the Central region led in telepathology services; however, by 2023, the Eastern region took over. Significant differences were observed in telepathology services between the Central and Western regions across the years, with annual average growth rates of 17.7% and 27.2%, respectively. Tertiary hospitals consistently reported higher median telepathology case volumes than secondary hospitals, with a statistically significant annual average growth rate of 26.2%.

**Table 1.** Distribution of telepathology services in different regions and different levels of hospitals.

Characteristics	Telepathology service volume per year, median (IQR)				*H*	*P* value
	2018	2019	2020	2023		
District						
East	36.5 (10-105)	80 (27.75-215)	31 (11.25-120.75)	359 (164.75-676.25)	4.55	.21
Center	93 (30-226)	141 (60-287)	148 (47-258)	210 (112.5-331.5)	10.11	.02
West	24 (2-180)	13 (2-123.5)	124 (60-776)	80 (36.75-146.75)	11.32	.01
Hospital grade						
Third-level	64 (5-292.25)	124 (60.5-245)	120 (25-274.5)	205 (109.75-420.25)	14.15	<.001
Second-level	50 (14.5-165)	55 (6.5-213)	80 (30-157)	100 (32.5-218)	2.15	.54
Total	51 (10-200)	95 (18.25-228.5)	100 (27.5-223.75)	200 (93-331.5)	17.61	.001

### The Operation and Charging Situation of Telemedicine in Hospitals That Carry Out Telepathology Services

Among the hospitals providing telepathology services in 2018, 2019, 2020, and 2023, the percentages with established telemedicine departments were 69.4% (75/108), 77.1% (54/70), 76.5% (52/68), and 72.9% (43/59), respectively. The median numbers of telemedicine staff allocated were 3 (IQR 2‐5) in 2018 and 2019, 4.5 (IQR 2‐7) in 2020, and 5 (IQR 2.5‐7) in 2023. Telemedicine operation modes included hospital self-operation, third-party operation, and mixed operation. Surveys over the 4 years showed that hospital self-operation was the primary mode, accounting for 67.59%, 50%, 67.65%, and 72.88%, respectively (Table S1 in Multimedia Appendix 4).

**Figure 2.**
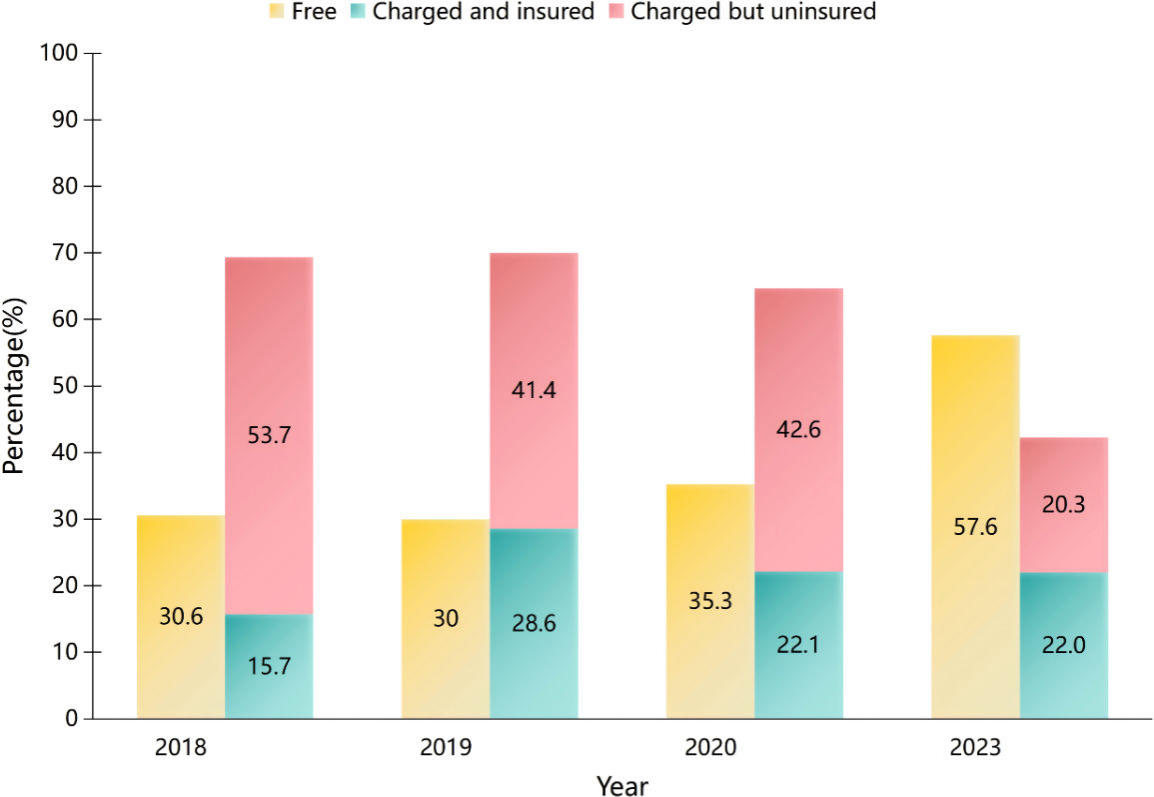
Proportions of hospitals providing telepathology services (free, charged and insured, charged but uninsured) in 2018‐2020 and 2023

In 2018, 2019, 2020, and 2023, among hospitals providing telepathology services, the percentages of free services were 30.6% (33/108), 30% (21/70), 35.3% (24/68), and 57.6% (34/59), respectively. For charged services reimbursed by medical insurance, the percentages were 15.7% (17/108), 28.6% (20/70), 22.1% (15/68), and 22% (13/59), respectively (Figure 2). Fee standards were based on 3 factors: administrative department guidelines, hospital-specific standards, and telemedical service provider criteria (Table S1 in Multimedia Appendix 4).

### Usage of Telepathology Among Pathologists

A total of 262 valid questionnaires were collected from pathologists, as shown in Table 2. The distribution of these pathologists by region was 69 from the eastern, 61 from the central, and 132 from the western regions. There were 129 (49.2%) males and 133 (50.8%) females with a mean age of 35.3 (SD 9.1, range 20-73) years. The dominant frequency was once a week, accounting for 61.1% (n=160). Combined, those who serviced once a week or 2 to 3 times a week made up 84.4% (n=221) of the total, whereas those servicing 4 to 5 times a week or more accounted for 15.6% (n=41). The average time spent on telepathology services was mainly 11 to 20 minutes (n=78, 29.8%) and 21 to 30 minutes (n=83, 31.7%).

**Table 2.** Demographic characteristics and telepathology usage among pathologists.

Characteristics	Participants, n (%)
District	
East	69 (26.3)
Center	61 (23.3)
West	132 (50.4)
Gender	
Male	129 (49.2)
Female	133 (50.8)
Age, y	
<30	76 (35.1)
30-39	106 (39.6)
40-49	60 (20.1)
≥50	20 (5.2)
Education	
Doctoral degree	13 (4.9)
Master’s degree	35 (13.3)
Bachelor’s degree	149 (56.8)
College diploma and below	65 (24.8)
Professional title	
Senior	23 (8.7)
Deputy senior	30 (11.4)
Intermediate	71 (27)
Primary and below	138 (52.6)
Frequency of telepathology use, time/wk	
1	160 (61.1)
2-3	61 (23.3)
4-5	23 (8.8)
6-7	3 (1.1)
≥8	15 (5.7)
Average duration of telepathology, min	
≤10	44 (16.8)
11-20	78 (29.8)
21-30	83 (31.7)
31-60	42 (16)
>60	15 (5.7)

### Pathologists’ Evaluation and Satisfaction With Telepathology

As shown in Table 3, 92.7% (n=243) of pathologists considered the system easy to operate, and the service process was convenient. Additionally, 95.5% (n=250) believed that telepathology could reduce patient burdens. The overall satisfaction rate with telepathology services was 95.8% (n=251), and 95% (n=249) of pathologists were willing to continue participation.

**Table 3.** The evaluation and satisfaction of telepathology.

Characteristics	Participants, n (%)
Convenience of system operation	
Very convenient	168 (64.1)
Convenient	75 (28.6)
Neutral	8 (3.1)
Inconvenient	9 (3.4)
Very inconvenient	2 (0.8)
Convenience of service process	
Very convenient	149 (56.9)
Convenient	94 (35.9)
Neutral	8 (3.1)
Inconvenient	10 (3.8)
Very inconvenient	1 (0.4)
The role of telepathology in reducing the medical burden of patients	
Very helpful	166 (63.4)
Helpful	84 (32.1)
Neutral	5 (1.9)
Unhelpful	4 (1.5)
Very unhelpful	3 (1.1)
Using intention of telepathology	
Very willing	153 (58.4)
Willing	96 (36.6)
Neutral	10 (3.8)
Unwilling	2 (0.8)
Very unwilling	1 (0.4)
Overall satisfaction	
Very satisfied	147 (56.1)
Satisfied	104 (39.7)
Neutral	6 (2.3)
Dissatisfied	4 (1.5)
Very dissatisfied	1 (0.4)

### Factors Influencing Pathologists’ Satisfaction and Use Intention of Telepathology

According to the univariate analyses shown in Tables2  3, 6 variables were associated with overall satisfaction (*P*<.25), and 5 variables were associated with use intention (*P*<.25) (Table 4). Multivariate ordinal logistic regression analysis revealed that 3 variables were included in the models for pathologists’ overall satisfaction and use intention, respectively. Both models met the proportional odds (parallel lines) assumption, with test results showing *P*=.83 for the satisfaction model and *P*=.44 for the usage intention model. Convenience of system operation and service processes significantly improved both overall satisfaction and willingness to use telepathology. Compared with an average service duration of ≤10 minutes per session, longer service times were associated with improved willingness to continue using the service. In addition, a moderate frequency of participation enhanced pathologists’ overall satisfaction.

**Table 4.** Ordinal logistic regression results of the influencing factors of the attitude toward telepathology among pathologists.

Variables	Overall satisfaction		Using intention	
	OR^a^ (95% CI)	*P* value	OR (95% CI)	*P* value
Threshold (ref=very satisfied/very willing)				
Very dissatisfied/very unwilling	—^b^	<.001	—	<.001
Dissatisfied/unwilling	—	<.001	—	<.001
Neutral	—	<.001	—	<.001
Satisfied/willing	—	.13	—	.19
Education (ref=doctoral degree)				
College diploma and below	2.53 (0.52-12.39)	.25	1.92 (0.41-8.91)	.41
Bachelor’s degree	1.26 (0.30-5.30)	.76	0.87 (0.21-3.53)	.85
Master’s degree	0.50 (0.11-2.28)	.37	0.36 (0.08-1.57)	.17
Professional title (ref=senior)				
Primary and below	1.26 (0.40-3.99)	.70	0.54 (0.17-1.70)	.29
Intermediate	1.48 (0.46-3.81)	.51	1 (0.31-3.23)	>.99
Deputy senior	0.89 (0.25-3.21)	.86	0.67 (0.19-2.39)	.54
Frequency of telepathology use (ref=≥8 time/wk)			—	—
1 time/week	0.26(0.06-1.13)	.07		
2-3 times/week	0.21 (0.04-0.96)	.045		
4-5 times/week	0.36 (0.06-1.98)	.24		
6-7 times/week	0.15 (0.01-2.56)	.19		
Average duration of telepathology (ref=≤10 min)				
≥61 min	4.03 (0.93-17.51)	.06	6.68 (1.48-30.23)	.01
31-60 min	1.98 (0.74-5.34)	.18	1.92 (0.75-4.94)	.18
21-30 min	1.76 (0.75-4.12)	.19	2.49 (1.09-5.67)	.03
11-20 min	1.78 (0.75-4.21)	.19	2.30 (1.01-5.26)	.049
Convenience of system operation (ref=Very convenient)				
Very inconvenient	6.21 (0.23-169.52)	.28	2.76 (0.12-64.33)	.53
Inconvenient	0.76 (0.08-7.50)	.81	0.70 (0.08-5.82)	.74
Neutral	0.06 (0.01-0.50)	.01	0.02 (0.00-0.18)	<.001
Convenient	0.44 (0.20-0.94)	.03	0.49 (0.23-1.04)	.06
Convenience of service process (ref=Very convenient)				
Very inconvenient	0.05 (0.00-7.92)	.25	0.08 (0.00-9.46)	.30
Inconvenient	0.07 (0.01-0.54)	.01	0.20 (0.03-1.32)	.09
Neutral	0.08 (0.01-0.70)	.02	0.25 (0.04-1.77)	.17
Convenient	0.22 (0.10-0.46)	<.001	0.29 (0.14-0.59)	.001

a OR: odds ratio.

b Not applicable.

### Patients’ Perceptions of Telepathology Service Quality

A total of 141 patients who had used telepathology services participated in the survey, as shown in Table 5. The distribution across eastern, central, and western provinces was 31.9% (n=45), 32.6% (n=46), and 35.5% (n=50), respectively. There were 64 males and 77 females, with a male-to-female ratio of 0.83:1. Ages ranged from 19 to 72 years, with a mean age of 33.5 (SD 10.5) years. Most patients held a bachelor’s degree (n=59, 41.8%), followed by an associate degree (n=50, 35.5%). Urban residents accounted for 61% (n=86) of patients. Employee medical insurance was the most common type of coverage (n=81, 57.5%), followed by the New Rural Cooperative Medical Insurance (n=38, 27%). Notably, 76.6% (n=108) of patients reported an average annual family income below 30,000 yuan (US $4335), which is lower than the national per capita disposable income of 39,218 yuan (US $5667) reported in the 2024 China Statistical Yearbook.

**Table 5.** Characteristics of the patients participating in telepathology.

Characteristics	Participants, n (%)
District	
East	45 (31.9)
Center	46 (32.6)
West	50 (35.5)
Age (y)	
<30	61 (43.3)
30-39	46 (32.6)
40-49	23 (16.3)
≥50	11 (7.8)
Gender	
Male	64 (45.4)
Female	77 (54.6)
Education	
Doctoral degree	2 (1.4)
Master’s degree	6 (4.3)
Bachelor’s degree	59 (41.8)
Associate degree	50 (35.5)
High school and below	21 (14.9)
Other	3 (2.1)
Average annual family income (¥)^a^	
<5000	42 (29.8)
5000-9999	26 (18.4)
10,000-14,999	17 (12.1)
15,000-19,999	7 (5)
20,000-24,999	9 (6.4)
25,000-29,999	7 (5)
30,000-49,999	8 (5.7)
≥50,000	25 (17.7)
Location	
Urban	86 (61)
Rural	55 (39)
Insurance	
Employee medical insurance	81 (57.5)
Medical insurance for urban residents	16 (11.4)
New rural cooperative medical insurance	38 (27)
Business insurance	2 (1.4)
No medical insurance	1 (0.7)
Other	3 (2.1)

a Exchange rate at the time of the study: US $1=¥6.92.

A total of 99.3% (140/141) of patients reported that the doctor thoroughly explained telepathology, and 96.5% (136/141) had signed the informed consent form, as shown in Table 6. All patients received diagnostic opinions from senior experts. Within 24 hours, 92.2% (130/141) received results; within 48 hours, 98.6% (139/141) received results. Only 1 patient (1/141, 0.7%) received results within 72 hours. Overall, 98.6% (139/141) of patients were satisfied with the waiting time. All 141 patients found telepathology convenient and beneficial for their condition. Regarding service cost, 63.1% (89/141) were charged, with 52 of 89 (58.4%) considering the charges very reasonable, 36 of 89 (40.5%) reasonable, and only 1 of 89 (1.1%) unreasonable.

**Table 6.** Telepathology service quality and patient satisfaction (N=141).

Characteristics	Participants, n (%)
Did doctor introduce telepathology in detail?	
Very detailed	97 (68.8)
Detailed	43 (30.5)
Not detailed	1 (0.7)
Did you sign the informed consent form?	
Yes	136 (96.5)
No	5 (3.5)
Convenience of telepathology service	
Very convenient	96 (68.1)
Convenient	45 (31.9)
Inconvenient	0 (0)
Have you received the expert diagnostic opinion?	
Yes	141 (100)
No	0 (0)
Waiting time (h)	
≤12	101 (71.6)
12.1-24	29 (20.6)
24.1-48	9 (6.4)
48.1-72	1 (0.7)
>72	1 (0.7)
Are you satisfied with the waiting time?	
Very satisfied	88 (62.4)
Satisfied	51 (36.2)
Dissatisfied	2 (1.4)
Is telepathology helpful for diseases?	
Very helpful	100 (70.9)
Helpful	41 (29.1)
Unhelpful	0 (0)
Is there a fee for this service?	
Yes	89 (63.1)
No	52 (36.9)
Is the fee reasonable? (n=89)	
Very reasonable	52 (58.4)
Reasonable	36 (40.5)
Unreasonable	1 (1.1)
Has medical expenses been saved?	
Yes	134 (95)
No	7 (5)
Willingness to recommend to others?	
Very willing	93 (66)
Willing	47 (33.3)
Unwilling	0 (0)
Very unwilling	1 (0.7)
Overall satisfaction	
Very satisfied	91 (64.5)
Satisfied	50 (35.5)
Dissatisfied	0 (0)

### Patient Satisfaction With Telepathology Services

The overall satisfaction rate was 100% (141/141), with 64.5% (91/141) reporting being “very satisfied”. Among patients, 95% (134/141) believed that telepathology could save medical expenses, and 99.3% (140/141) were willing to recommend it to others. The main reasons for recommendation included convenience (91/140, 65%), access to quality medical services (22/140, 15.7%), cost saving (11/140, 7.9%), time saving (6/140, 4.3%), and necessity (1/140, 0.7%).

## Discussion

### Principal Results

Diagnosis consistency between WSI and light microscopy has been confirmed, and telepathology is increasingly adopted worldwide [26]. It is widely used for remote primary diagnosis, second-opinion consultations, quality assurance, education, and research [27]. In China, the government has actively promoted telepathology to address the shortage of pathologists in primary health care institutions. However, although most medical institutions in China have implemented telemedicine systems, the utilization rate has remained below 20%, as highlighted at the 2019 subforum of the Global Health Forum of Boao Forum for Asia: Innovation for Health—The Future of Internet Medicine [28]. The actual use of telepathology in Chinese hospitals remains systematically understudied. Although annual surveys by TIPC and NTCC provide cross-sectional snapshots of telemedicine development, they are largely descriptive and lack dedicated analysis of telepathology or longitudinal assessment. To address this gap, this study provides the first systematic examination of telepathology development in China through long-term trend analysis, regional comparisons, and dual perspectives from both clinicians and patients. Based on these findings, targeted recommendations are proposed to inform policy-making and support the sustainable advancement of telepathology in China.

Regarding telepathology infrastructure construction, the highest proportion is networks, while the lowest is digital slide scanners. This is because invited experts only need to provide diagnostic suggestions based on WSI uploaded by the inviting institutions, eliminating the need for slide scanners. From 2018 to 2023, the number of telepathology service cases in China grew steadily, with the median per hospital increasing from 51 to 200 cases, reflecting an average annual growth rate of 31.4%. However, surveys revealed that the proportion of hospitals with established telepathology systems exceeded the proportion of those actually providing services each year. The ratio of service implementation to system construction ranged from 73.8% (59/80) to 82.3% (70/85), a trend consistent with the reported service utilization rates of 72.3% (60/83) to 89.1% (74/83) in the China CNCTPS telepathology platform report [21]. Hitchcock [29] pointed out that the limited implementation of telepathology in developing countries primarily stems from a lack of standardized equipment, inconsistent image formats, and insufficient training for pathologists. Practices in Nigeria [16] further highlighted the significant time cost associated with image scanning and uploading (5‐10 min per slide for scanning and 10‐30 min for uploading to the cloud), which has become a critical bottleneck affecting service efficiency. Research by Indian scholars [17] also indicated that challenges such as the large file sizes of WSI, insufficient transmission speeds, and the need for security protection of patient data and images during internet transmission are key factors limiting the application of telepathology. In response to these challenges, various technical approaches have been proposed in the academic community. Emerging technologies such as IoT [30], blockchain-based digital identity authentication [31], and aspect-oriented programming [32] have been proven effective in reducing data transmission latency, enhancing medical information security, and improving system interoperability. These technologies offer feasible solutions for addressing the challenges of cross-institutional secure retrieval of large WSI files. Therefore, promoting the integrated application of these technologies in telepathology holds significant potential for increasing service utilization rates in the future. Moreover, policy and standardization improvements are crucial. Health authorities should promote WSI standardization, clarify cross-institutional licensing rules, and include telepathology in insurance reimbursement.

From a regional distribution perspective, the development of telepathology varies across different areas. Similarly, a national survey in Ireland [33] indicated that the advancement of telemedicine differs significantly across regions, with multiple regional networks established in the east, while development in the west predominantly consisted of isolated links. Our study reveals that the eastern region experienced the fastest growth, with an average annual growth rate of 58%, likely attributable to its more abundant medical resources, stronger economic foundation, and the advantages of policy piloting and early implementation. From 2018 to 2020, the central region reported the highest median number of telepathology diagnostic cases, which may be associated with its lower urbanization rate and relatively scarce rural healthcare resources. According to the China Statistical Yearbook, the urbanization rate in the eastern region was 68.5%, compared with 56.8% in the central region and 54.1% in the western region. Additionally, data from the China Health Statistical Yearbook indicate that from 2018 to 2020, the central region consistently had the lowest number of health technicians per thousand rural population. These factors likely contributed to a higher demand in the central region. To address regional disparities in China’s telepathology services, a systematic policy intervention is essential. This includes: (1) Investment: establishing a dedicated fund for central/western regions and integrating telepathology into medical insurance; (2) Operations: implementing unified technical standards and regional diagnostic centers with performance incentives; (3) Capacity: training local pathology technicians and physicians.

The dominant frequency of telepathology usage among pathologists in China was once a week, accounting for 61.1% (160/262). In contrast, a national survey in the United Kingdom revealed that telepathology was rarely used on a weekly or monthly basis [34]. Meanwhile, surveys in South Africa showed that 11% (8/71) used telepathology weekly [35], and a study in Switzerland reported a 31% (42/134) weekly usage rate for telepathology [36]. The average time spent on telepathology services is mainly 11 to 20 and 21 to 30 minutes (161/262, 61.5%), which aligns with the reported average duration of telemedicine services in China [37]. In contrast, a study from the United Kingdom reported that the time taken for telepathology diagnosis averaged 3.9 minutes [38]. Pathologists’ acceptance of telepathology demonstrates a strong global consensus. In our study, 95.8% (251/262) of participating pathologists reported overall satisfaction, with 95% (249/262) willing to continue its use. This aligns with international findings: a Saudi Arabian study noted only 1.6% (1/64) had no intention to use telepathology [39]; a UK study involving 18 pathologists found 88.89% (16/18) were likely to continue digital reporting [40]; and a Japanese study showed 70% (16/23) were interested in the system for consultations [41]. Notably, our survey revealed a higher rate of strong intention to use (153/262, 58.4%) compared to the Saudi study (23/64, 35.9%) [39]. This trend extends beyond pathology, as evidenced by a global survey of urologists from 58 countries, where 80.9% (197/244) intended to continue using telemedicine in clinical practice [42]. When analyzing the influencing factors of satisfaction and using intention among pathologists, this study demonstrated that the convenience of system operations and service process had a significant positive impact on pathologists’ satisfaction and willingness to use telepathology. Additionally, longer service durations were also associated with positive impact, consistent with prior research on telemedicine services [37]. However, no significant associations were found between pathologists’ satisfaction or willingness to use telepathology and factors such as gender, age, education level, or professional title. This is consistent with the findings from a survey of 64 pathologists in Saudi Arabia [39]. The manual navigation tools required for digital slides and the single-layer 2D nature of WSIs demand careful scrutiny by pathologists to ensure diagnostic accuracy. Shorter duration of telepathology may compromise diagnostic precision. Conversely, an intuitive system interface and streamlined service workflow facilitate smoother telepathology diagnosis, thereby enhancing pathologists’ engagement and platform retention. The research suggests that improving pathologists’ engagement in telepathology depends on optimizing both system design and workflow. To achieve this, medical institutions should: (1) enhance system usability and streamline the consultation process; and (2) reasonably schedule telepathology sessions based on workload and diagnostic quality requirements.

While research on telemedicine from an end-user perspective is extensive, studies focusing on telepathology user attitudes remain limited. This may be because telepathology often does not require direct patient involvement. Nevertheless, we surveyed 141 telepathology patients to assess their experiences and satisfaction. Among them, 92.2% (130/141) and 98.6% (139/141) received diagnostic results within 24 and 48 hours, respectively, surpassing the rates from our previous analysis of Henan Province’s telepathology network (64%, 14,835/23,167 and 82.9%, 19,201/23,167) [21] and exceeding those reported by the Québec Telepathology Network in Canada [43]. All patients expressed satisfaction, aligning with the high patient satisfaction reported in other telemedicine surveys in Canada (248/272, 91%) [44], Norway [45], Argentina (116/116, 100%) [46], and Africa [47]. Higher than the survey in India, which indicated that 78% (39/50) of patients with cancer expressed interest in future telemedicine opportunities [48], 99.3% (140/141) were willing to recommend telepathology to others, citing high-quality service, time savings, and cost savings as key reasons. Based on the findings of this study and international comparative data, telepathology services have demonstrated clear and significant advantages in diagnostic turnaround time and patient satisfaction. Therefore, it is recommended that in future public education and service promotion, the core messages of “high quality (expert diagnosis), high efficiency (rapid response), convenience (localized diagnosis and treatment)” and “high satisfaction (patient endorsement)” be precisely communicated. This approach aims to enhance patient awareness and trust, thereby encouraging patients to more proactively choose telepathology services when faced with diagnostic options.

### Limitations

This study analyzes the construction and application of telepathology in China from 2018 to 2023 through a nationwide survey, focusing on digital slide scanners, telepathology software, and network connectivity. It also evaluates the progress of telepathology services across hospitals of varying levels and regions. As the first detailed nationwide report on telepathology in China, this study uniquely examines perspectives from both pathologists and patients, covering usage, satisfaction, and influencing factors analysis. The findings provide a comprehensive understanding of telepathology in China and offer actionable recommendations for its enhanced application.

Although our findings offer valuable insights into telepathology in China, this study has several limitations. The sample was based on voluntary participation rather than a longitudinal cohort follow-up, and the significant shift in policy focus toward internet hospitals may have reduced engagement with this telemedicine-specific survey, contributing to the annual decline in sample size. The survey was conducted through TIPC, the national academic organization for telemedicine, and administered via the NTCC website, with participating hospitals spanning most regions. However, the online nature of the survey may have introduced selection bias. The survey spanned from 2018 to 2023, a period marked by significant advancements in telemedicine and a shift toward internet-based healthcare, driven by national policies. Policy shifts and potential survey fatigue contributed to a decline in valid responses each year. Additionally, the surveys for 2021 and 2022 were disrupted due to the pandemic. Despite these limitations, the survey encompassed a sufficient number of medical institutions across China, and data analysis revealed a clear growth trend in telepathology development. Therefore, while these limitations exist, their impact on the study’s conclusions is likely minimal. Besides, in-depth interviews with surveyed pathologists and patients are still lacking to fully understand the barriers of telepathology, so as to put forward more reasonable suggestions.

Given that this study is based on a comprehensive national survey in which the items related to telepathology were relatively general, future research will involve the design and implementation of a specialized telepathology survey. This survey will categorize questions according to service types—such as routine histopathological diagnosis and intraoperative frozen section consultation—to obtain more granular and in-depth data. This approach aims to comprehensively and thoroughly reveal the actual clinical application landscape and differentiated needs of telepathology. Additionally, we will further explore the perspectives of pathologists and patients towards telepathology through semi-structured interviews, which can help to devise corresponding strategies for the better development of telepathology in China.

### Conclusion

This study provides the first systematic examination of telepathology development in China through long-term trend analysis, regional comparisons, and dual perspectives of both clinicians and patients. The findings indicate significant growth in the construction and application of telepathology in China from 2018 to 2023, with high levels of acceptance from both pathologists and patients. Factors such as the convenience of system operation, smoothness of service processes, frequency of use, and average duration per session were significantly associated with pathologists’ satisfaction and willingness to use telepathology. To enhance the adoption rate of telepathology services, targeted recommendations are proposed around a synergistic “technology-institution-promotion” framework: at the technological level, integrating cloud computing and artificial intelligence to improve system usability; at the institutional level, establishing dedicated funds for central and western regions, promoting the inclusion of telepathology in medical insurance reimbursement, and refining cross-institutional licensing and performance incentive policies; at the promotional level, accurately communicating its core values of “high quality, high efficiency, convenience, and high satisfaction” to the public to encourage proactive patient choice. Through multidimensional systemic interventions, the technological advantages of telepathology can be translated into sustainable primary healthcare service capabilities.

## Supplementary material

10.2196/83514 Multimedia Appendix 1 Map of China Showing the Division of Eastern, Central, and Western Regions.

10.2196/83514 Multimedia Appendix 2 Distribution of the survey coverage areas in China from 2018 to 2023.

10.2196/83514 Multimedia Appendix 3 Survey on the development of telemedicine in Chinese hospitals.

10.2196/83514 Multimedia Appendix 4 Table S1. The operation and charging situation of telemedicine in hospitals that carry out telepathology services.

10.2196/83514 Checklist 1 CHERRIES checklist.

## Abbreviations

CHERRIES: Checklist for Reporting Results of Internet E-Surveys
NTCC: National Telemedicine Center of China
TIPC: Telemedicine Informationization Professional Committee of China
WSI: whole-slide image
