# Supplementary figures and images for: Telepathology Development and Stakeholder Perspectives in China: Cross-Sectional Survey
Source: JMIR Med Inform. 2026 Mar 16;14:e83514. doi: 10.2196/83514 (PMC12991195; doi:10.2196/83514)

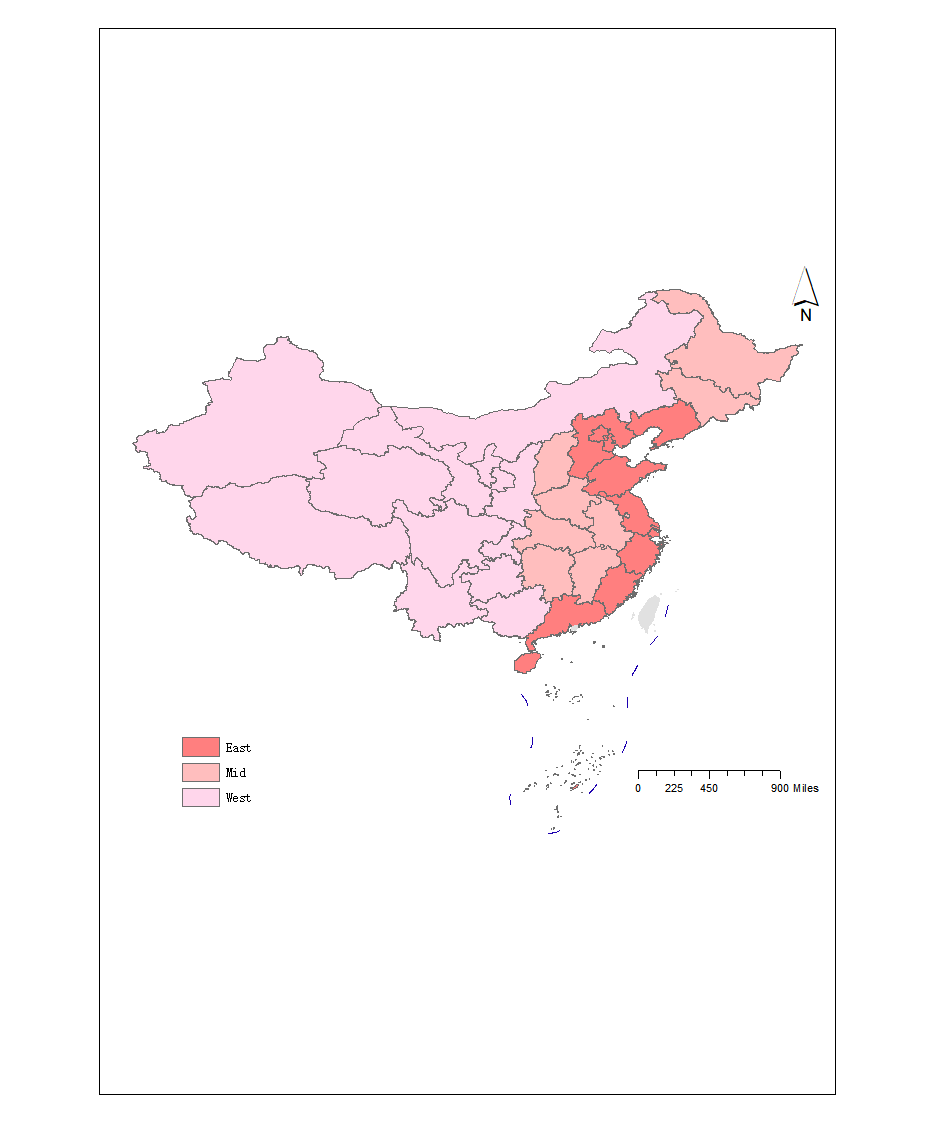

Supplement: Multimedia Appendix 1 [file medinform-v14-e83514-s001.png]
