# Supplementary material for: Telepathology Development and Stakeholder Perspectives in China: Cross-Sectional Survey
Source: JMIR Med Inform. 2026 Mar 16;14:e83514. doi: 10.2196/83514 (PMC12991195; doi:10.2196/83514)
Supplement: Multimedia Appendix 3 [file medinform-v14-e83514-s003.docx]

**Survey on the Development of Telemedicine in Chinese Hospitals**

(Questions related to telepathology)

**Introduction**

The survey was organized by the Telemedicine Informationization Professional Committee of China (TICP) and the National Telemedicine Center of China (NTCC), with the aims to fully understand the development of telemedicine in China, and provide a scientific basis for the further development of telemedicine. We sincerely hope all participating hospitals provide accurate information. We will take any valuable information provided seriously and pay attention to protecting the hospital information, ensuring that the information will not be leaked.

**Notes on filling the questionnaire**

The person filling in the questionnaire should be an employee responsible for telemedicine work.

**Basic information**

1. Hospital name ：
2. Hospital Grade

①tertiary hospitals ②secondary hospitals ③primary hospitals

**Construction and Implementation of Telepathology**

1. Which of the following remote medical equipment does your hospital currently possess (multiple choices)?

①Remote video conferencing terminal

②Remote ECG acquisition terminal (standard ECG)

③Remote ECG monitor (dynamic ECG)

④Remote mobile ward rounding cart

⑤Remote education recording and broadcasting equipment

⑥Remote surgical demonstration equipment

**⑦Digital slide scanner**

⑧Other

1. What telemedicine systems have been established in your hospital currently (multiple choices)?

①Teleconsultation system

②Remote education system

③Remote ward rounds system

④Remote surgery teaching system

**⑤Telepathology system**

⑥Remote image diagnosis system

⑦ Remote ECG diagnosis system

⑧Tele-outpatient clinic system

⑨Remote emergency care system

⑩Remote Chinese medicine system

⑪Other

1. What kind of network does your hospital use for telemedicine?

①Virtual private network (VPN, constructed based on wired network) ②Public Internet(excluding VPN and wireless networks)

1. What types of telemedicine applications have been developed in your hospital (multiple choices)?

①Teleconsultation, the annual business volume: cases per year

②Remote education, the annual business volume: cases per year

**③Telepathology, the annual business volume:**  **cases per year**

④Tele-electrocardiogram, the annual business volume: cases per year

⑤Tele-diagnosis of medical images, the annual business volume: cases per year

⑥Remote surgery teaching

⑦Remote intensive care

⑧Tele-outpatient clinic

⑨Remote nursing

⑩Remote bidirectional referral

⑪Remote ward rounds

⑫ Remote chronic disease management

⑬Remote emergency care

⑭Other

**The operation and charging situation of telemedicine**

1. What is the management mode of telemedicine service in the hospital？

①Self-management mode

②Partial entrustment mode

③Completely entrust mode

1. Has the hospital set up a special operation and management department for telemedicine business?

①Having already setup a management department

②No

1. How many staff are engaged intelemedicine in the hospital?

Please fill in the number of staff

1. What are the charge items included in the telemedicine business currently carried out by your hospital (multiple choices)?

①Teleconsultation

②Remote education

③Remote ward rounds

④Remote surgery teaching

**⑤Telepathology**

⑥Tele-diagnosis of medical images

⑦Tele-electrocardiogram

⑧Remote nursing

⑨Remote chronic disease management

⑩Other

1. What is the standard for setting the charges for your hospital's telemedicine services?

①The hospital sets its own pricing standards.

②Administrative departments establish uniform pricing standards.

③Charges are determined based on the pricing standards of the counterpart telemedicine service provider.

1. Is the cost of telemedicine services in your hospitals covered by medical insurance for reimbursement?

①Yes

②No

**Thankyou for your cooperation!**

**Survey on the usage and perceptions of telemedicine**

(Doctors)

**Introduction**

The survey was organized by the Telemedicine Informationization Professional Committee of China (TICP) and the National Telemedicine Center of China (NTCC), targeting medical personnel who have participated in telemedicine services.

This questionnaire is conducted anonymously. When filling out this questionnaire, please read each question carefully and answer truthfully based on your actual experiences. We will take the information you provide seriously and pay attention to protecting the privacy of the information you fill in.

If you have any questions while filling out the questionnaire, please feel free to contact the survey team. Contact numbers: 0371-67966298 (Miss. Li), 0371-67966213 (Mr. Jiang).

**Basic information**

1. Hospital name ：
2. Your Department

①Internal Medicine ②Surgery ③Gynecology ④ Pediatrics

⑤Pathology ⑥ Imaging ⑦ Electrocardiography ⑧ Other

1. Your Gender

① Male

② Female

1. Your age is ________ years old.
2. Your Occupation Category

① Doctor

② Nurse

③ Medical Technician

④ Other

1. Your Professional Title

① Intermediate or below

② Associate Senior

③ Senior

1. Your Highest Degree

① Doctoral

② Master's

③ Bachelor's or below

**Telemedicine Service Situation**

1. Which telemedicine service projects do you primarily participate in?

① Remote Consultation

② Remote Pathology Diagnosis

③ Remote Imaging Diagnosis

④ Remote Electrocardiography Diagnosis

⑤ Other

1. How often do you participate in telemedicine services on average per week?

① Once a week

② 2-3 times a week

③ 4-5 times a week

④ 6-7 times a week

⑤ 8 times or more

1. What is the average duration of your participation in telemedicine services each time?

① Less than 10 minutes

② 11-20 minutes

③ 21-30 minutes

④ 30-60 minutes

⑤ More than 60 minutes

1. How convenient is the operation of the telemedicine system you use?

① Very convenient

② convenient

③ Inconvenient

④ Very inconvenient

1. Is the process of the telemedicine services you participate in convenient?

① Very convenient

② Convenient

③ Inconvenient

④ Very inconvenient

1. Do you think telemedicine services can help reduce the medical burden on patients?

① Very helpful

② Helpful

③ Slightly unhelpful

④ Very unhelpful

1. Are you willing to continue participating in telemedicine services?

① Very willing

② Willing

③ Unwilling

1. What is your overall satisfaction with telemedicine services?

① Very satisfied

② Satisfied

③ Dissatisfied

**Thankyou for your cooperation!**

**Survey on the usage and perceptions of telemedicine**

(Patients)

**Introduction**

In order to better protect the rights and interests of patients receiving telemedicine services and continuously improve the quality and level of telemedicine services, the Telemedicine Informationization Professional Committee of China (TICP) and the National Telemedicine Center of China (NTCC) has organized this survey.

The target respondents of this questionnaire are patients who have received telemedicine services, and it can be filled out with the assistance of the patient's accompanying family members.

This questionnaire is conducted anonymously. When filling out this questionnaire, please read each question carefully and answer truthfully based on your actual experiences. We will take the information you provide seriously and pay attention to protecting the privacy of the information you fill in.

If you have any questions while filling out the questionnaire, please feel free to contact the survey team. Contact numbers: 0371-67966215 (Miss. Li), 0371-67966286 (Miss. He).

**Basic information**

1. Your location of medical visit ：
2. Your Gender

① Male

② Female

1. Your age is ________ years old.
2. Your household registration type is ( )

① Urban

② Rural

1. Your medical insurance type is ( )

① Employee Medical Insurance

② Medical Insurance for Urban Resident

③ New Rural Cooperative Medical Insurance

④ Business Insurance

⑤ No medical insurance

⑥ Other

1. Your highest level of education is ( )

① High school or below

② Associate degree

③ Bachelor’s degree

④ Master’s degree

⑤ Doctoral degree

⑥ Other

1. Your annual household income per capita is ( )

① Below 5,000

② 5,000–9,999

③ 10,000–14,999

④ 15,000–19,999

⑤ 20,000–24,999

⑥ 25,000–29,999

⑦ 30,000–49,999

⑧ 50,000 or above

**Telemedicine Service Quality Survey**

1. Before receiving telemedicine services, did the medical staff provide you with a detailed introduction to telemedicine services? ( )

① Very detailed

② Detailed

③ Not detailed

1. Before receiving telemedicine services, did the medical staff ask you to sign an informed consent form? ( )

① Yes

② No

1. How long did you wait from submitting the application to the completion of the telemedicine service? ( )

① ≤12 hours

② 12.1–24 hours

③ 24.1–48 hours

④ 48.1–72 hours

⑤ >72 hours

1. Your satisfaction level with the waiting time mentioned in 10 is ( )

① Very satisfied

② Satisfied

③ Dissatisfied

1. After the consultation, did your attending doctor provide you with feedback on the diagnosis and treatment opinions from the experts at the higher-level hospital? ( )

① Yes

② No

1. Is telemedicine helpful for diseases? ( )

① Very helpful

② Helpful

③ Unhelpful

1. Were you charged for the telemedicine services you received? ( ) (If you choose ①, answer B7; if you choose ②, skip to 16)

① Yes

② No

1. How reasonable do you think the charges for the telemedicine services are? ( )

① Very reasonable

② Reasonable

③ Unreasonable

1. Do you think telemedicine services saved you medical expenses? ( )

① Yes

② No

1. How convenient do you think the telemedicine services are for you? ( )

① Very convenient

② Convenient

③ Inconvenient

1. Your overall satisfaction with the current telemedicine services is ( )

① Very satisfied

② Satisfied

③ Dissatisfied

1. Your willingness to recommend telemedicine services to other patients is ( )

① Very willing

② Willing

③ Unwilling

If willing, the reason you are willing to recommend telemedicine services is: ________

If unwilling, the reason you are unwilling to recommend telemedicine services is: ________

**Thankyou for your cooperation!**
