# Supplementary material for: Telepathology Development and Stakeholder Perspectives in China: Cross-Sectional Survey
Source: JMIR Med Inform. 2026 Mar 16;14:e83514. doi: 10.2196/83514 (PMC12991195; doi:10.2196/83514)
Supplement: Multimedia Appendix 4 [file medinform-v14-e83514-s004.docx]

**Table S1.** The operation and charging situation of telemedicine in hospitals that carry out telepathology services

| Characteristics | In 2018  (N=108) | In 2019  (N=70) | In 2020  (N=68) | In 2023  (N=59) |
| --- | --- | --- | --- | --- |
| **Telemedicine departments, (n, %)** | | | | |
| Yes | 75(69.4) | 54(77) | 52(76) | 43(73) |
| NO | 33(30.6) | 16(23) | 16(24) | 16(27) |
| **The median number of telemedicine staff, Median (IQR)** | | | | |
|  | 3(2-5) | 3(2-5) | 4.5(2-7) | 5(2.5-7) |
| **The operation of telemedicine,(n, %)** | | | | |
| hospital self-operation | 73(67.6) | 35(50) | 46(68) | 43(73) |
| mixed | 26(24.1) | 35(50) | 19(28) | 16(27) |
| out-party operations | 9 (8.3) | 0(0) | 3(4) | 0(0) |
| **Whether there is a charge,(n, %)** | | | | |
| Yes | 75(69.4) | 49(70) | 44(65) | 25(42) |
| formulated by the health administrative departments | 38(35.2) | 37(53) | 19(28) | / |
| drafted by hospitals | 14(13) | 5(7) | 5(7) | / |
| according to the standards of the telemedicine service institution | 23(21.3) | 7(10) | 20(29) | / |
| No | 33(30.6) | 21(30) | 24(35) | 34(58) |
| **Whether the fee is included in medical insurance,(n, %)** | | | | |
| Yes | 17(15.7) | 20(29) | 15(22) | 13(22) |
| No | 58(53.7) | 29(41) | 29(43) | 12(20) |
